# Design of dot-blot hybridization assay for simultaneous detection of *Campylobacter jejuni* and *Campylobacter coli*: a preliminary study

**DOI:** 10.1097/MS9.0000000000001558

**Published:** 2023-12-06

**Authors:** Bita Bakhshi, Saeed Shams, Niloofar Rezaie, Mahdieh Ameri Shah Reza

**Affiliations:** a Department of Bacteriology, Faculty of Medical Sciences, Tarbiat Modares University; b Department of Microbiology, Pasteur Institute of Iran, Tehran; c Cellular and Molecular Research Center, Qom University of Medical Sciences, Qom, Iran

**Keywords:** *Campylobacter coli*, *Campylobacter jejuni*, Dot-blot hybridization, recombinant plasmid

## Abstract

**Objectives:**

Campylobacters are a major cause of gastroenteritis worldwide. These are fastidious in culture and false negative results are seen in many clinical laboratories. Among molecular methods, the dot-blot technique is widely used for a variety of purposes, especially diagnostics. So, the authors aimed to detect *C. jejuni* and *C. coli* simultaneously using a dot-blot assay.

**Methods:**

After evaluating the bioinformatics studies, a *cadF*-conserved fragment was selected for the design of primers and probe. DNAs from standard strains and a recombinant plasmid, prepared in this study, were used to assess the technique. The specificity of the method was also surveyed using DNAs from other enteric bacteria. The limit of detection was evaluated by recombinant plasmid and different concentrations of the designed probe.

**Results:**

A 95-bp fragment of *cadF* was selected, and in silico analysis studies showed that it is conserved between both species. Also, the non-specific annealing of the primers and probe with other bacteria was not seen theoretically. The technique with recombinant plasmid as well as DNAs of standard strains created black spots on the membrane, confirming that the probe was correctly synthesized. No non-specific reactions with other bacterial species were observed (specificity=100%). The limit of detection of the test was determined to be 50 µg/ml.

**Conclusions:**

This is the first study to simultaneously detect two important pathogens in the *Campylobacter* genus and was able to detect *C. jejuni* and *C. coli* with acceptable sensitivity and specificity.

## Background

Highlights

- This is the first to develop a dot-blot assay for detecting *C. jejuni* and *C. coli*.
- This technique was able to detect the bacteria with acceptable sensitivity.
- The designed dot-blot assay showed a high specificity.

The *Campylobacter* (*C.*) spp. are spiral, Gram-negative, microaerophilic bacteria and consist of 27 species and 8 subspecies that can asymptomatically colonize in microbial habitats including intestinal tracts of most mammals and birds^1–3^. The disease in humans is called campylobacterosis, which is mostly caused by *C. jejuni* (~90%) and *C. coli* (~10%). This infection, which is mainly seen in children, is known as a zoonotic disease and can be transmitted through animals such as chickens, pigs, cattle, and sheep and their products^4–9^.

Epidemiologically, campylobacterosis is mainly found in developing countries and is endemic in Africa, Asia, and the Middle East, but is also on the increase in North America, Europe, and Australia^10,11^. In many countries, the isolation of *Campylobacter* spp. in humans is 3–4 folds higher than the number of *Salmonella* spp. and *E. coli*.

Infection is usually self-limiting, but symptoms can include watery diarrhoea (may be bloody), abdominal cramps, fever, vomiting, etc. However, severe and prolonged conditions may occur in immunocompromised patients^12,13^. In addition, the subsequent *Campylobacter* infection may be associated with reduced growth in children, Guillain-Barré syndrome, irritable bowel syndrome, inflammatory bowel disease, and reactive arthritis^14,15^. Co-infection of this genus with other microorganisms has also been reported^16^.

Various methods such as culture, serology, and molecular assays are used to diagnose campylobacters, but each has its advantages and disadvantages^17–21^. Culture is the gold standard for diagnosing bacteria, but it is time-consuming and viable but non-culturable (VBNC) status can occur under stress and adverse cultivation conditions, so its sensitivity can be reduced^22,23^. Serological tests are other techniques that may be used in some cases to diagnose *Campylobacter* infections. Unfortunately, cross-reactions between bacteria are seen in these methods^24^.

Molecular assays are more sensitive than other methods that can quickly identify the target and can replace conventional methods. In some methods, such as polymerase chain reaction (PCR), the contamination of the genome, particularly from previous reactions, or enzymatic inhibitors can lead to false positive or false negative results, respectively. Also, although the specificity of conventional PCR confirms the size of visualized bands on an agarose gel, further specificity of reactions needs to perform hybridization by blotting techniques^25^.

The development of dot-blot hybridization has been done for the detection of some bacteria with high sensitivity and specificity^26,27^. Thus, the aim of this study was to advance a dot-blot technique for detecting both *C. jejuni* and *C. coli*.

## Material and methods

Theoretical and laboratory studies (2022–2023) of this work were carried out at Qom University of Medical Sciences, Qom, Iran, and Tarbiat Modares University (TMU), Tehran, Iran, respectively.

### Selection of the target gene fragment for the design of probe and primers

The whole sequence of the *C. jejuni* (accession no.: CP048764.1, CP028911.1, CP022076.1, LN831025.1, CP010906.1, CP020045.1, LR134497.1, CP038862.1, CP063085.1, CP001876.1, OU532558.1, CP006006.1, HG428754.1, CP048767.1) and *C. coli* (accession no.: KC575115.1, CP109813.1, CP007181.1, CP017025.1, KC876748.1, NZ_CP092026.1, GL405235.1, CP006702.2, NZ_CP091644.1, CP092025.1) genomes were obtained from the GeneBank (http://www.ncbi.nlm.nih.gov/) using the CLC Sequence Viewer 8.0 software (CLC bio, Aarhus, Denmark). Among the diagnostic genes, *cadF* was selected as the target gene, and its full sequence was isolated from the whole genomes. Then, the alignment of *cadF* genes between *C. jejuni* and *C. coli* was performed to identify a conserved region, which was used as the probe in the dot-blot technique. In addition, the primers on this fragment were designed as follows: F, 5′- GATAATCGTTATGCACCAGGGA-3′ and R, 5′- ACATCAGAATAATGCTCTAACCCA-3′, and purchased from TAG Copenhagen (Denmark). Theoretically, the specificity of the selected probe fragment and the designed primers was confirmed using BLAST (https://blast.ncbi.nlm.nih.gov/Blast.cgi) and Primer-BLAST (https://www.ncbi.nlm.nih.gov/tools/primer-blast/), respectively.

### Standard bacterial strains and culture

In this study, the standard strains stored from the bacterial collection of the Medical Bacteriology Department of TMU were used. *C. coli* ATCC 43478 and *C. jejuni* ATCC 29428 were cultured on Brucella agar (Merck, Germany) supplemented with 5% sheep blood and incubated at ~42°C for 24–48 h under microaerophilic atmospheric conditions. Other standard strains used in the specificity test were also cultured on relevant media and all were used after confirmation by Gram staining and biochemical tests.

### DNA extraction and PCR

The genome was extracted from the bacteria using the conventional boiling method. The PCR reaction was carried out in a 25 μl mixture, containing 10 μl of Master Mix 2X (Ampliqon, Denmark), 1 μl of each primer (10 pmol/ μl), 5 μl of the extracted genome from *C. jejuni* ATCC 29428, and 8 μl of sterile distilled water. PCR was carried out under the following conditions: genome denaturation step at 95°C/3 min (1 cycle), followed by 32 cycles of denaturation at 94°C/30 sec, annealing at 57°C/30 sec, and extension at 72°C/30 sec, and then additional extension step at 72°C/10 min in a thermocycler (Eppendorf, Hamburg, Germany). PCR products were electrophoresed on 1% agarose gel.

### Preparation of recombinant plasmid as the positive control

TA cloning was done using the PCR production of the *cadF* gene of *C. jejuni* ATCC 29428. The fragment was ligated into a pTZ57R/T vector according to the manufacturer’s instructions (InsTAclone PCR Cloning Kit Fermentas). *E. coli* TOP10F’, as competent cells, were transformed with the ligation products and incubated at 37°C for 24 h on LB medium containing tetracycline and ampicillin (Merck). To confirm the cloning, colony-PCR, enzymatic digestion, and sequencing (Macrogen) were performed^28^.

### Synthesizing of the probe

To synthesize the probe (5′- ACATCAGAATAATGCTCTAACCCAAATTCTAATTGATCAAGCCAAAAATCGTCAAAATGATAACCAAGTCTAATCCCTGGTGCATAACGATTATC-3′), the extraction of the PCR products, as the template, from the gel was carried out using the QIAquick Gel Extraction Kit (Qiagen, Valencia). 3- Dig-Probes were synthesized using a Dig Oligonucleotide 3-End Labelling Kit according to the manufacturer’s instructions (Roche).

### Dot-blot hybridization

For initial hybridization setup, first, 1 μl of recombinant plasmid and genomes extracted from *C. jejuni* ATCC 29428 and *C. coli* ATCC 43478 were spotted onto a positively charged nylon membrane and dried at room temperature. Next, the membrane was soaked in denaturation buffer (1.5M NaCl, 0.5N NaOH) for 30 min, and then in neutralization buffer (0.5M Tris-HCl, 1.5M NaCl) for 30 min. At the end of the incubation, the membrane was washed twice in a 2× saline–sodium citrate (SSC) for 30 min. The nylon membrane was baked at 80°C for 2 h. Prehybridization was performed in a hybridization solution for 30 min at the calculated optimal temperature of 35°C prior to the hybridization with the probe overnight in the same buffer in a hybridization oven. Following hybridization, the membrane was washed twice with 2× SSC for 5 min and then treated in 0.5× SSC for 15 min at room temperature. Then, the strip was first incubated in a blocking solution (10% blocking powder in maleic acid buffer) for 30 min and subsequently, 20 ml anti-DIG antibodies (150 mU/ml Anti-Digoxigenin-AP in blocking solution) were added for 30 min at room temperature on a shaker. The membrane was washed twice in washing buffer for 15 min on a shaker. Finally, the results of the test were detected with 5-bromo-4-chloro-3-indolyl phosphate–nitro blue tetrazolium (BCIP-NBT).

### Specificity and limit of detection (LOD) of the assay

The specificity of the assay was identified using extracted DNAs from other enteric non-*Campylobacter* strains, for example *Vibrio cholerae* ATCC 14035, *Listeria monocytogenes* ATCC 7644, *Aeromonas hydrophila* ATCC 7966, *Yersinia enterocolitica* ATCC 23715, *Salmonella typhimurium* ATCC 14028, and *Enterobacter cloacae* PTCC 1798. The limit of detection of the method was evaluated using concentrations of 500, 250, 100, 50, and 5 µg/ml of the designed probe.

## Results

### Bioinformatics analysis of target gene

**Figure 1:**
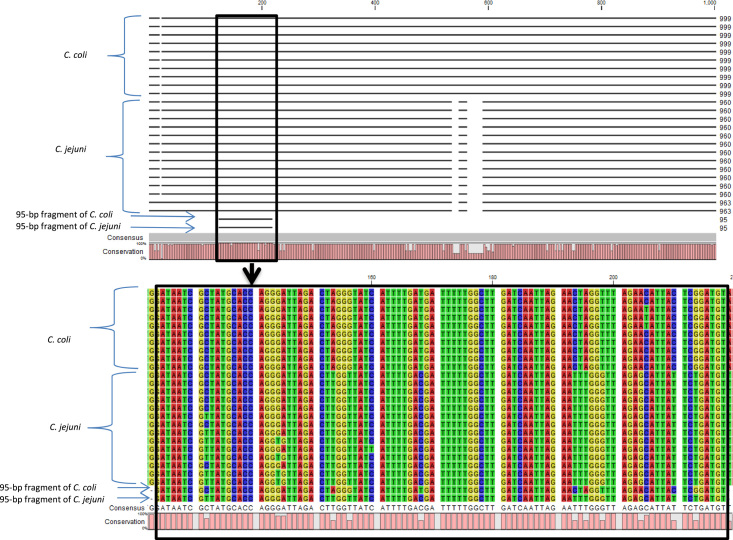
Alignment result of *cadF* gene of *C. jejuni*/*C. coli* strains and position of the 95-bp fragment using CLC Sequence Viewer software.

The full length of the *cadF* gene in *C. jejuni* and *C. coli* was 960 and 999 bp, respectively. Based on in silico analyses, a 95-bp fragment of the *cadF* gene was selected in the study. This target showed GC content of about 34–37% for both bacteria and is located at position 124–218. Although there are minor mismatches in the length of the selected fragment, this region was conserved among *C. jejuni* and *C. coli* strains. Therefore, the probe and primers designed for this region were able to simultaneously identify these bacteria (Fig. 1). In addition, theoretically, non-specific annealing of probe and primers with other bacteria was not observed (data not shown).

**Figure 2:**
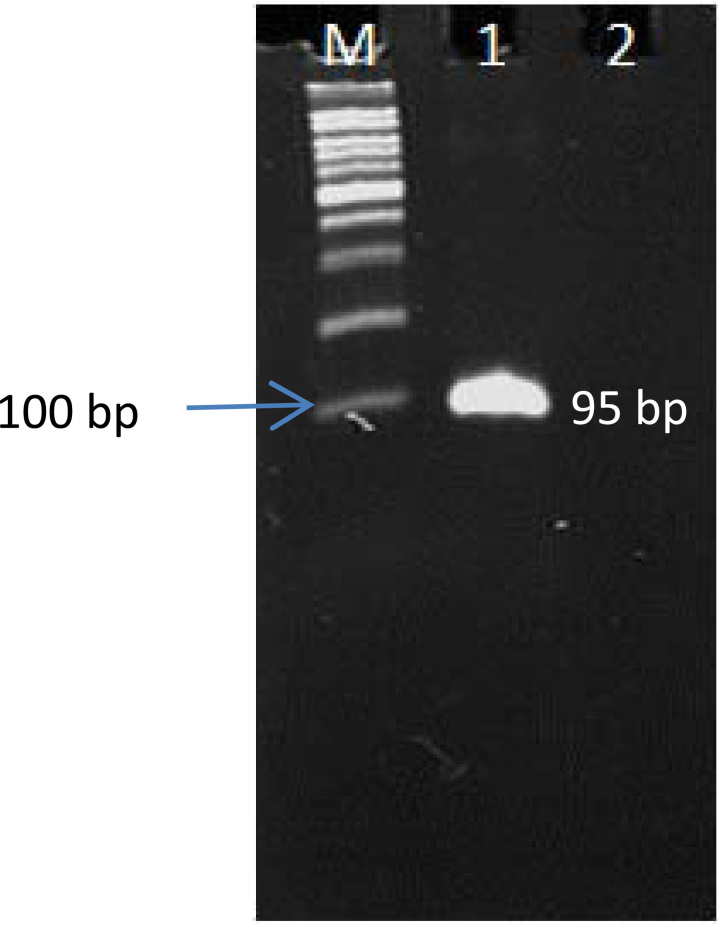
Electrophoresis of polymerase chain reaction products of the 95-bp fragment. M: 100 bp; lane 1: *C. jejuni* ATCC 29428; lane 2: negative control.

**Figure 3:**
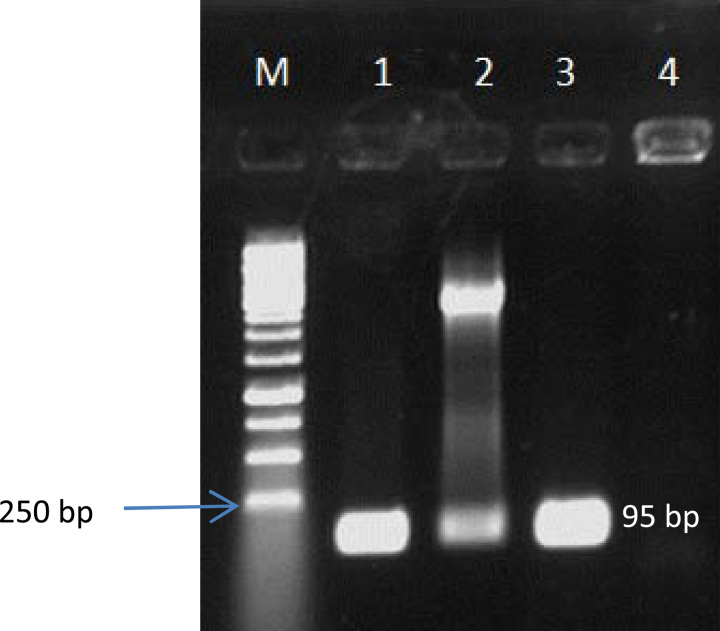
Electrophoresis of colony- polymerase chain reaction (PCR) products and enzymatic digestion. M: 1kb marker; lane 1: colony-PCR from host *E. coli*; lane 2: double digestion of plasmid; lane 3: PCR of *C. jejuni* ATCC 29428 (positive control); and lane 4: negative control.

Also, the PCR result showed that the target fragment was amplified well, and its product was appropriate for probe synthesis (Fig. 2). Colony-PCR, enzymatic digestion (Fig. 3), and sequencing (data not shown) results indicated that TA cloning was done very well, and the fragment was correctly inserted into the plasmid. Therefore, the recombinant plasmid was suitable for use as a positive control.

### Dot-blot hybridization

**Figure 4:**
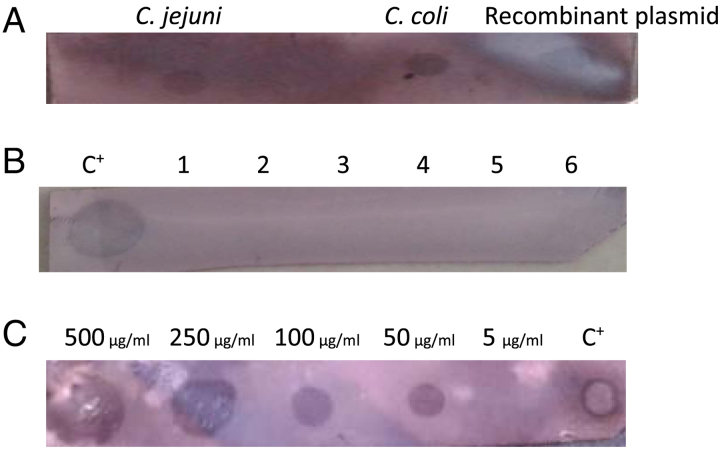
Specificity and LOD of dot-blot hybridization technique. A. Initial setup of hybridization with recombinant plasmid, *C. jejuni* ATCC 29428, and *C. coli* ATCC 43478; B. Specificity test: Recombinant plasmid (C^+^), *Listeria monocytogenes* ATCC 7644, *Vibrio cholerae* ATCC 14035, *Aeromonas hydrophila* ATCC 7966, *Enterobacter cloacae* PTCC 1798, *Yersinia enterocolitica* ATCC 23715, *Salmonella typhimurium* ATCC 14028; C. LOD: No spot was seen at the concentration of 5  µg/ml.

The dot-blot process with the recombinant plasmid and DNAs extracted from standard strains of *C. jejuni* and *C. coli* created black spots on the nylon membrane, confirming that the probe was correctly synthesized. In the evaluation of the specificity of the method, no reaction between the probe and other bacterial genomes was identified (specificity=100%). The LOD of the test was determined at 50 µg/ml and no visible spot was detected with a 5 µg/ml concentration. As the concentration decreased, the size of the spot also became smaller (Fig. 4 A-C).

## Discussion

Diarrhoea caused by *Campylobacter* spp., similar to other gastrointestinal bacteria as well as microbial agents, has increased as an important disease in recent decades in both developed and developing countries^10,29–31^. Campylobacteriosis is usually endemic in parts of Asia, especially in Iran, and mainly affects children^32,33^. The culture and biochemical tests are still one of the most widely using methods for the identification of *Campylobacter* species in clinical laboratories. Unfortunately, because isolation of these bacteria from clinical samples is difficult and time-consuming due to their fastidious growth characteristics, false negative results may be reported.

On the other hand, molecular methods for diagnosing infectious diseases are expanding. Today, the use of these techniques such as PCR, biosensors, etc. is being developed because they are faster, simpler, and more reliable than culture^23^. However, due to special equipment, these are not available everywhere.

Due to diagnostic problems, there is no accurate epidemiological report about this disease in most parts of the world, and as a result, the design of a suitable diagnostic method is of interest to researchers. Thus, in this study, a dot-blot hybridization was designed to detect both *C. jejuni* and *C. coli* simultaneously.

There are a few studies regarding the use of the dot-blot method to diagnose infectious diseases. In a study by Niu and colleagues in 2012, PCR and dot-blot techniques were used to detect the *ibeA* gene in *E. coli*. Their results showed that dot-blot assay is a rapid and highly accurate method for screening clinical samples^34^. In Sun’s study in 2005, variable regions of the 16S gene of *E. coli*, *P. mirabilis*, *K. oxytoca*, and *P. aeruginosa* were selected for designing oligonucleotide probes in a sensor assay. They showed a rapid and species-specific hybridization consistent with other similar studies^35^. In Ho’s study, the combined PCR-Reverse dot-blot assay was used to identify some bacterial intestinal pathogens, including *B. cereus, C. botulinum, C. perfringen, S. aureus, L. monocytogenes, Salmonella* spp., *E. coli* O157:H7, *Shigella* spp., *V. cholerae*, *V. parahaemolyticu*s, *Y. enterocolitica*, and *Brucella* spp., in blood samples. The results of this study showed that this technique with high specificity can be used to identify pathogens in other samples such as stool or food^36^.

However, there are different results in this field. For example, in a 2003 study conducted in Finland, PCR primers and hybridization probes were designed for the 16S rRNA genes of six species of bacteria, Their results showed that real-time PCR was easier and faster with more sensitivity for detection in comparison to dot-blot hybridization^37^.

There are a few genes that are used for the detection of *Campylobacter* spp. such as *cadF*, *asp*, *hipO*, *ceuB*-*C*, *lpxA,* etc^20^. Among the diagnostic genes, *cadF* was selected in the present study. This gene showed a high percentage of conservation among *C. jejuni C. coli* strains. By theoretically evaluating the entire length of the gene, a conserved fragment (95 bp) was selected to detect both *Campylobacter* species. Also, in the experimental investigation, the gene fragment was completely specific to C*. jejuni* and *C. coli*, and non-specific binding of the probe and primers was not observed. So, this result showed the high specificity of the technique. In addition, our dot-blot test was able to detect the target at the lowest concentration of 50 μg/ml.

Only a few studies have been published regarding the design of the dot-blot method for the detection of *Campylobacter*. In a 1988 study conducted, the dot-blot hybridization method was used for *Campylobacter* diagnosis using three non-radioactive probes (2 biotinylated probes and 1 sulphonated probe) with the ^32^P-labelled probe. In their study, the lowest concentration detected for the ^32^P, biotin-DNA and sulphonated probes was 100 pg, and for photobiotin-DNA probe was 10 ng^38^. Fontanot and colleagues study used two molecular methods including dot-blot and PCR to identify *Campylobacter* spp. The dot-blot method had a sensitivity of 25 ng to detect DNA extracted from the *Campylobacter* reference strains using a probe labelled with digoxigenin^39^. Jensen and colleagues also report real-time PCR and hybridization assays for the detection of thermophilic *Campylobacter* spp. in pig faecal samples. Their results showed that colony-blot hybridization using the *hipO* gene probe can provide the specific isolation of *C. jejuni* from pigs^40^.

## Conclusion

As a preliminary study, this is the first to simultaneously detect two important enteropathogenic bacteria in the *Campylobacter* genus, including *C. jejuni* and *C. coli*. According to the results of the present study, dot-blot hybridization can be a simple method with high sensitivity and specificity to identify the infection caused by these species. In addition, this technique seems to be more useful than other molecular assays. However, to prove this point, further studies on pure clinical isolates and even direct evaluation of this technique on clinical samples are recommended. The design of this method with other diagnostic genes of the target bacteria is also suggested.

### Limitation

Due to limitations in clinical sampling, hybridization evaluation was not performed on these samples.

## Ethical approval

This study was approved by the ethics committee of Qom. University of Medical Sciences (Code No.: IR.MUQ.REC.1402.071).

## Consent

None.

## Sources of funding

Qom University of Medical Sciences and Tarbiat Modares University.

## Author contribution

S.S. and B.B. developed and supervised the work; M.A.S.R. and N.R. performed the experiments; S.S., B.B., and N.R. drafted the manuscript; All authors reviewed the manuscript. Also, all authors read and approved the final manuscript.

## Conflicts of interest disclosure

The authors declare no conflicts of interest.

## Research registration unique identifying number (UIN)

None.

## Guarantor

Saeed Shams.

## Data availability statement

I confirm if any datasets generated during and/or analyzed during the current study are publicly available, available upon reasonable request.

## Provenance and peer review

None.

## Acknowledgements

The authors thank the Research Council of Qom University of Medical Sciences and Tarbiat Modares University for supporting this project.
